# A Comprehensive Analysis of Non-Thermal Ultrasonic-Assisted Extraction of Bioactive Compounds from Citrus Peel Waste Through a One-Factor-at-a-Time Approach

**DOI:** 10.3390/molecules30030648

**Published:** 2025-02-01

**Authors:** Matthew A. Xuereb, Georgios Psakis, Karen Attard, Frederick Lia, Ruben Gatt

**Affiliations:** 1 Metamaterials Unit, Faculty of Science, University of Malta, MSD 2080 Msida, Malta; matthew.a.xuereb@um.edu.mt (M.A.X.); georgios.psakis@um.edu.mt (G.P.); 2 Institute of Applied Sciences (IAS), The Malta College of Arts, Science and Technology (MCAST), PLA 9032 Paola, Malta; karen.attard@um.edu.mt; 3 Centre for Molecular Medicine and Biobanking, University of Malta, MSD 2080 Msida, Malta

**Keywords:** *Citrus sinesis*, citrus peel waste valorization, ultrasonic-assisted extraction, one-factor-at-a-time approach, bioactive compound recovery

## Abstract

Food waste presents a critical environmental and economic challenge across Europe. In the Mediterranean region, the agricultural industry generates considerable quantities of citrus fruits, leading to significant byproduct waste, which remains underutilized. To help address this, this study explored the valorization of orange peel waste using non-thermal ultrasonic-assisted extraction (UAE) and a one-factor-at-a-time experimental design to investigate the effects of nine chemical and physical UAE parameters. The goal was to identify ideal operational ranges for each parameter using several responses (bioactive compound recovery, antioxidant activity, and radical scavenging activity), thus elucidating the most influential UAE factors and their role in co-extracting various classes of natural compounds. The key findings revealed that the polarity and ionic potential of the extraction medium, tuned through ethanol:water or pH, significantly influenced both the chemical profile and bioactivity of the extracts. Notably, citric acid and citrates appeared to stabilize co-extracted compounds. Lower solid-to-liquid ratios increased yields, while particle sizes between 1400 and 710 µm enhanced phenolic recovery by approximately 150 mg/L GAE. In contrast, increases in pulse, probe diameter, immersion depth, and extraction time led to degradation of bioactive compounds, whereas the maximal amplitude improved phenolic acid recovery by up to 2-fold. Collectively, these insights provide a foundation for optimizing non-thermal UAE to valorize orange peel waste.

## 1. Introduction

The citrus processing industry generates a significant amount of waste byproducts, accounting for nearly 50% of the fresh fruit mass. Of this total waste mass, 50 to 55% by weight consists of peel, while 20 to 40% is made up of seeds [1,2,3,4,5]. To provide perspective, the global citrus processing industry produces approximately 10 million tonnes of waste annually [5]. These byproducts are rich in beneficial bioactive compounds like polyphenols, essential oils, pectin, dietary fibers, carotenoids, and ascorbic acid [2,3,6,7,8,9,10]. These constituents exhibit superior antioxidant properties compared to the edible portions of the fruit and provide various health benefits. Their high biological activity and antioxidant properties contribute to antiviral, anti-inflammatory, anti-carcinogenic, cardiovascular-protective, and neuroprotective effects [9,11,12,13,14,15,16,17,18]. Moreover, citrus byproducts can be converted into bio-based products like enzymes, biofuels, and biopolymers [6,7,19,20]. Citrus peel waste, which amounts to approximately 60 to 65% by weight of the waste from processed citrus fruits [10,21], holds promise for applications in the food, cosmetics, and pharmaceutical industries for producing pectin, dietary fibers, and flavoring agents [5,20]. It could also contribute to the development of functional foods and serve as a natural preservative [5,9]. The valorization of citrus byproducts presents opportunities for creating functional foods, advancing sustainability, and establishing new economic pathways in the bioeconomy. Nevertheless, further investigation is necessary to scale up and commercialize such extraction processes.

Ultrasonic-assisted extraction is a highly efficient method for extracting bioactive compounds from citrus peels, offering advantages such as reduced solvent consumption, lower extraction temperatures, and improved product quality [22,23,24,25,26,27]. The extracted compounds, including hesperidin and vanillin, have potential applications in the food and pharmaceutical industries [20,28]. This technique utilizes acoustic cavitation to enhance mass transfer and accelerate extraction through the mechanisms of (i) cellular disruption, (ii) high solvent penetration, and (iii) particle size reduction, resulting in higher yields and shorter extraction times compared to traditional methods [24,25,29,30]. Several critical variables influence UAE performance, including extraction time, temperature, ultrasound power, solvent type, and solid-to-solvent ratio, with optimal conditions varying for different bioactive compounds [22,29,30,31,32,33,34,35,36,37,38,39,40]. Additionally, UAE has been found to increase mass transfer rates of antioxidants from citrus peel and pomace by 30% [28]. Overall, UAE presents a fast, efficient, and economical method for valorizing citrus peel waste. Optimizing UAE parameters can significantly enhance extraction efficiency, making it a promising technique for sustainable industrial applications [6,24,31,41].

Orange peel contains a variety of antioxidant compounds, such as flavonoids, phenolic acids, and carotenoids [20,21,30,42,43,44]. By means of HPLC and ultra-high performance liquid chromatography (UPLC), researchers have been able to identify different types of flavonoids present in orange peel, including polymethoxylated flavones, C-glycosylated flavones, and O-glycosylated flavones [45,46,47,48,49,50]. The main phenolic compounds found in both ripe and unripe orange peels are quercitrin, rutin, and quercetin; compounds which are found in higher quantities in ripe peels, thus exhibiting higher antioxidant activity [50]. By optimizing the extraction process, researchers have been able to obtain high concentrations of bioactive compounds, such as hesperidin, polyphenols, ascorbic acid, and carotenoids [10,17,21,25,30,40,43,44,46,51,52]. Among citrus fruits, orange peel has been found to have the highest antioxidant properties, followed by lemon and grapefruit peels. This activity is mainly attributed to the flavanone glycosides hesperidin and naringin [53]. The extraction of phenolic compounds from orange peel utilizing thermal and non-thermal UAE has been investigated in various studies [30,38,40,43,45,51,54]. For instance, in the research by Khan et al. [43], temperature, power, and ethanol concentration were examined, whereas Montero-Calderon et al. [30] focused on optimizing the extraction time, power, and ethanol concentration. Razola-Diaz et al. [54] implemented a Box–Behnken design with four independent factors: ethanol, time, amplitude, and pulse, allowing the authors to improve the recovery of phenolics by up to 60% when compared to conventional extractions. Although UAE has been employed to optimize the recovery of polyphenols from waste orange peel, to our knowledge, no single work has systematically investigated the nine pertinent parameters (namely extraction time, solid-to-solvent ratio, particle size, ultrasound-pulse, ultrasound-amplitude, ethanol concentration, pH, ultrasonic probe head diameter, and depth of probe immersed in the solution) for their effects on bioactive compound yields and antioxidant activity. Such a thorough investigation is difficult to achieve using traditional multi-response techniques, because the large number of variables often overshadows the less commonly investigated variables such as pH, immersion depth, probe diameter, and particle size. Furthermore, multi-response approaches are typically designed to examine the combined effect of variables on a single outcome, making it challenging to analyze the co-extraction of natural compound classes (phenolic acids, flavonoids, glycosides, and organic acids).

**Figure 1:**
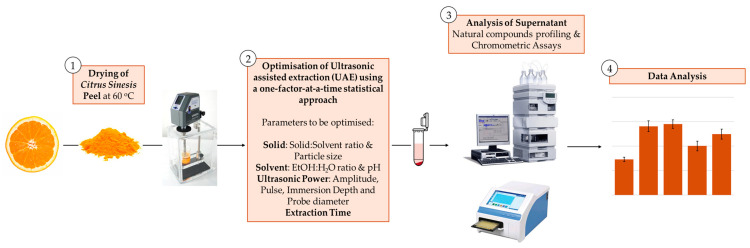
Outline of the one-variable-at-a-time experimental design implemented in the UAE from *Citrus sinesis* peel.

In view of these considerations, this study adopted a one-factor-at-a-time approach to independently analyze nine key chemical and physical variables associated with the non-thermal UAE of waste orange peel, identifying optimal operating ranges for phenolic compound recovery, the co-extraction of the various natural compounds classes, antioxidant activity, and radical scavenging activity. These evaluations were performed though both chromometric assays and HPLC (Figure 1). More specifically, this study aimed to

- Provide a comprehensive screening of nine specific extraction parameters, each tested independently using a one-factor-at-a-time approach, to assess their individual effects on bioactive compound yields and antioxidant activity from waste orange peel.
- Highlight the conditions (or their ranges) that favor maximizing the extraction of particular classes of compounds, such as flavonoids, phenolic acids, and glycosides.

It is hoped that this comprehensive analysis will not only underscore the efficacy of this green extraction technology but also offer crucial information on a number of overlooked variables, thereby facilitating high-throughput, industrial-scale food waste valorization.

## 2. Results and Discussion

### 2.1. Solid Parameters

#### 2.1.1. Solid-to-Feed or Solid-to-Solvent Ratio

**Figure 2:**
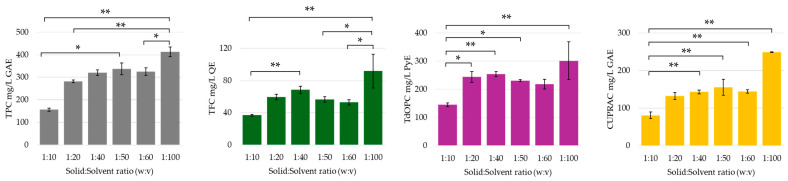
Total phenolic content (TPC), total flavonoid content (TFC), total orthodiphenol content (TdOPC), and CUPRAC activity of UACPE extracts prepared with various solid:solvent ratios. Values are normalized per unit mass of orange peel used. Horizontal bars annotated with two asterisks indicate statistically significant differences between pairs with a 95% confidence level. Horizontal bars annotated with one asterisk indicate statistically significant differences between pairs with a 90% confidence level.

An increase in solvent-to-feed or solvent-to-solid ratio generally leads to a decrease in the concentration of the extract, while simultaneously improving the overall yield, as the amount of starting material used is greater [26,55,56,57,58,59]. This trend was also observed in this study, where a decrease in solute-to-solvent ratio (lower solute content) resulted in a higher yield of phenolic compounds and enhanced cupric ion reducing antioxidant capacity assay (CUPRAC) antioxidant activity (see Figure 2). This observation can be attributed to the significant influence of the solute-to-solvent ratio on the mass transfer dynamics, as a larger volume of solvent facilitates the acceleration of diffusion processes, since it becomes more difficult to reach the solubility or saturation limit of the phenolic compounds. A lower solute content also results in a decreased mixture density, which in turn, facilitates the propagation of ultrasonic waves, while simultaneously diminishing the attenuation of ultrasonic power. Consequently, the lowered density allows for more efficient energy transfer, which in turn, enhances extraction efficiency. Furthermore, the diminished density of the mixture amplifies the cavitation effect, thereby further aiding the extraction process. For this parameter, the concentrations of phenolic compounds and organic acids obtained through HPLC-UV are provided in the Supplementary Information (Figure S7).

#### 2.1.2. Particle Size

**Figure 3:**
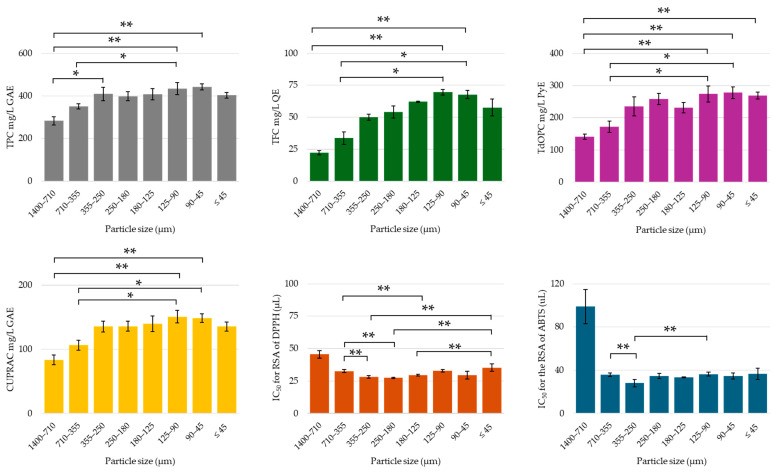
TPC, TFC, TdOPC, CUPRAC, and RSA of UACPE extracts prepared with various particle size ranges. Horizontal bars annotated with two asterisks indicate statistically significant differences between pairs with a 95% confidence level. Horizontal bars annotated with one asterisk indicate statistically significant differences between pairs with a 90% confidence level. Significant differences between the RSA response of the 1400–710 µm particle size and all the other parameter settings were omitted for clarity.

The size of particles is a significant factor in UAE, as smaller particles typically enhance extraction efficiencies [26,35,39,60,61,62,63]. This study demonstrated notable variations in the yield of phenolic compounds and the corresponding CUPRAC activity when citrus peel was ground to different particle sizes and subjected to UAE (see Figure 3). In the case of this study, the optimal particle size for extracting phenolic compounds from citrus peel appeared to be below 710 μm, depending on the specific target compound and the form of plant material. A significant difference was observed between the 1400 to 710 µm size range and smaller size ranges, particularly the 125 to 90 µm and 90 to 45 µm ranges. This difference was also reflected in the radical scavenging activity (RSA) of the UACPE extracts against DPPH radicals and ABTS radical cations. Specifically, the IC_50_ value for the 1400 to 710 µm range was statistically significantly higher compared to other extracts (see Figure 3), correlating the increased polyphenolics content with improved radical scavenging potential. The graphs in Figure 3 suggest that as the particle size decreased, the levels of TPC, TFC, TdOPC, and CUPRAC initially increased, likely due to an increased surface area to volume ratio (SA:V), which enhanced extraction. However, when the particles became very small (under 100 µm), they tended to clump together in the liquid, reducing the effective surface area exposed to the solvent and lowering the extraction efficiency. Additionally, smaller particles can hinder the cavitation process, as the increased number of particles in the medium scatters and absorbs ultrasound energy, weakening the cavitation effect. Furthermore, a higher frequency of smaller particles may result in energy loss, as the microjets formed during cavitation are less likely to collide with plant material, due to a lower probability of collision. On the other hand, the lower phenolic content at the larger particle sizes (1400–710 μm) was likely due to the reduced surface area-to-volume ratio, which limited the efficiency of acoustic cavitation and mass transfer. Cavitation and the microjets formed by collapsing bubbles directly affect the particle surface, enhancing extraction. However, with smaller particles, cavitation can become less effective as more ultrasound energy is absorbed by the greater number of particles, rather than contributing directly to the extraction process. For this parameter, the concentrations of phenolic compounds and organic acids obtained through HPLC-UV are provided in the Supplementary Information (Figure S8).

### 2.2. Solvent Composition

#### 2.2.1. Ethanol Concentration (%)

**Figure 4:**
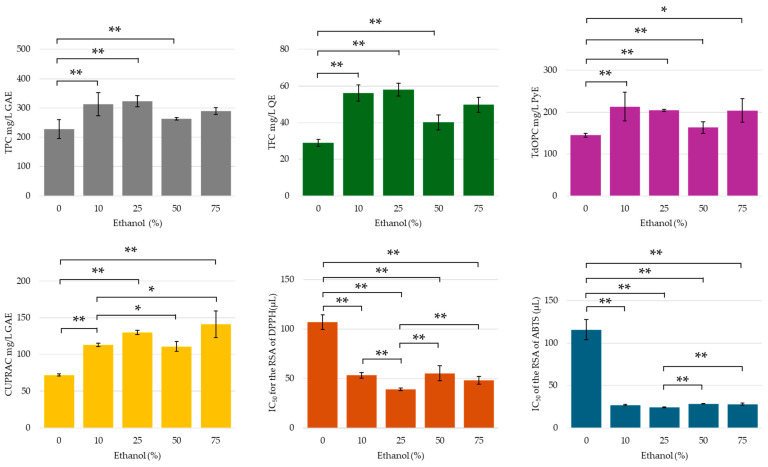
TPC, TFC, TdOPC, CUPRAC, and RSA of UACPE extracts prepared with various ethanol concentrations. Horizontal bars annotated with two asterisks indicate statistically significant differences between pairs with a 95% confidence level. Horizontal bars annotated with one asterisk indicate statistically significant differences between pairs with a 90% confidence level.

The intracellular distribution of phenolic compounds is predominantly governed by their solubility, which is intrinsically linked to their polarity [64]. Therefore, the selection of an appropriate extraction solvent must correspond with the solubility profiles of the compounds of interest. Solvents such as methanol and ethanol possess significantly lower polarity than water, which facilitates the solubility and diffusion of phenolic compounds. The findings indicated a substantial increase in phenolic content, as well as corresponding CUPRAC activity, when the ethanol concentration was increased from 0% to 10% (see Figure 4). This increase may be attributed to the higher concentration of less polar and/or hydrophobic compounds, likely resulting from the decreased dielectric constant of the solvent mixture and reduced co-extraction of highly polar sugars and organic acids. No significant differences were observed between the yields of phenolics, flavonoids, and orthodiphenols between extracts prepared with 10 and 25% ethanol solutions, suggesting that the changes in the extraction medium’s polarity did not significantly influence the extraction efficiency. When the extraction solvent was composed of 50% ethanol and 50% water, a decline in the extracted phenolic content was observed. This reduction may be attributed to the dehydration and structural collapse of plant cells, as well as the denaturation of cell wall proteins with the increased ethanol concentration [65,66,67], which together complicated the extraction of phenolic compounds. On the other hand, extracts at 75% ethanol showed higher phenolic and flavonoid contents compared to those prepared at 50% ethanol. Apart from the possible improved solubility of certain phenolic compounds at higher ethanol concentrations, the efficiency of denaturation of the storage sites of specific polyphenolic compounds within the plant material may also be a contributing factor. These results are also reflected in the radical scavenging potential of the extracts, whereby extractions conducted with 25% ethanol attained the lowest IC_50_ value for both radical and radical cation scavenging (see Figure 4). Extractions conducted in aqueous conditions had a significantly higher IC_50_ value, which may be explained by the lowest phenolic and flavonoid yields from this series of extracts.

**Figure 5:**
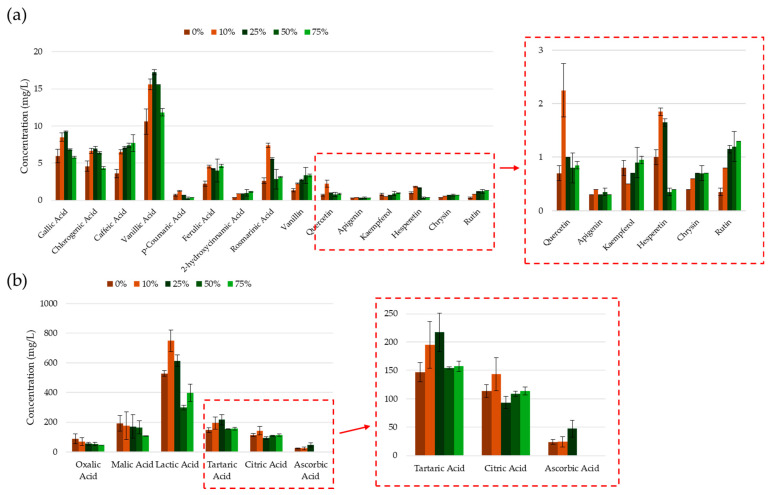
Concentrations of (**a**) phenolic compounds and (**b**) organic acids in the UACPE extracts prepared at various ethanol concentrations. Chromatograms for phenolic compounds and organic acids obtained through HPLC-UV are provided in the Supplementary Information.

The HPLC analysis of the corresponding UACPE extracts revealed a U-shaped trend in the extracted concentration of several phenolic compounds with increasing ethanol concentration. Specifically, the extracted concentration of these compounds increased, peaking between 10% and 25% ethanol, followed by a decline at 50 to 75% ethanol (see Figure 5). This result suggests that, while many phenolic acids and flavonoids exhibited a poor solubility in water and an increased solubility in ethanol (or other organic solvents) [68,69,70,71,72,73], a decrease in the polarity of the extraction solvent mixture did not ensure a more optimal extraction. Conversely, some phenolic compounds deviated from this general trend, showing a steady increase in the extracted concentration with rising ethanol levels, reaching their maximum concentration at 75% ethanol, something which may be linked to the denaturation of their storage location in the plant cell. These compounds included rutin (a glycoside formed from the flavonol quercetin and the disaccharide rutinose), the flavonoid chyrsin, vanillin (a benzaldehyde derivative of salicylaldehyde with a methoxy group at position 3), and the hydroxycinnamic acids: caffeic acid, ferulic acid, and 2-hydroxycinnamic acid.

The extraction of phenolic compounds was also accompanied by the co-extraction of various organic acids. In general, the pH of the extraction medium was found to increase as the aqueous content decreased, with the exception of the 10% ethanol extract. The pH values of the extracts were 4.74, 4.46, 5.03, 5.60, and 5.70, corresponding to the increasing ethanol concentrations used to prepare the extracts. The lower pH observed for the 10% ethanol extract may have resulted from the extraction of higher concentrations of phenolic acids (see Figure 5a), as well as tartaric, lactic, and citric acid. A key factor which may have contributed to the two dissimilar trends explained above for different phenolic compounds is the co-extraction of organic acids (see Figure 5b). Notably, tartaric, lactic, and citric acid follow the same general trend as the majority of phenolic compounds discussed earlier. Their co-extraction in high concentrations may stabilize the phenolic compounds through electrostatic or ionic interactions. Furthermore, the increased ionic potential of the extract solution may enhance mass transfer dynamics and solubility, thereby improving recovery [70,71,73,74]. Additionally, the conjugate base forms of ascorbic acid may play a vital role in the stabilization of phenolic compounds through various mechanisms [75,76,77,78,79]. This is particularly relevant, as ascorbic acid was absent in the 50% and 75% ethanol extracts, which exhibited a generally lower yield of phenolic compounds.

#### 2.2.2. pH

**Figure 6:**
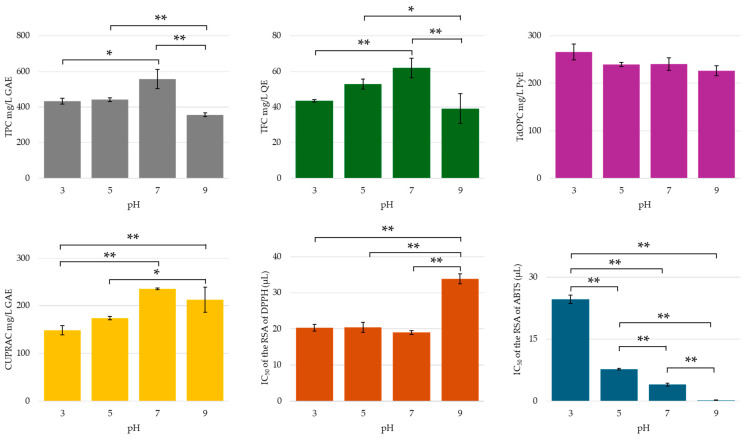
TPC, TFC, TdOPC, CUPRAC, and RSA of UACPE extracts prepared with various phosphate pH buffers in a 50:50 (*v*:*v*, %) mixture with ethanol. Horizontal bars annotated with two asterisks indicate statistically significant differences between pairs with a 95% confidence level. Horizontal bars annotated with one asterisk indicate statistically significant differences between pairs with a 90% confidence level.

The pH of extraction solvents plays a crucial role in determining both the yield and composition of phenolic compounds derived from various botanical sources [80,81,82,83,84]. However, to our knowledge, this has not yet been investigated in UAE studies focusing on the recovery of phenolic compounds from dried orange peel. The efficacy of the extraction process is influenced by the pH of the extraction medium, as it modifies the ionic strength, which in turn affects the solubility of the compounds and their interactions with the sample matrix [74,80,82,84,85]. The results indicated that the total phenolic and flavonoid content in the UACPE extracts increased significantly as the initial extraction buffer solution pH shifted from 3 to 7, followed by a decline at pH 9 (see Figure 6). This decline may be attributed to the prolonged application of ultrasonic energy at alkaline pH, potentially leading to the breakdown of the ionized or partially ionized phenolics and flavonoids. In contrast, the impact of pH on the levels of orthodiphenolic compounds was found to be negligible, with no statistically significant differences. In the CUPRAC assay, a positive trend was observed, plateauing at pH 7 and 9, as no statistically significant difference was noted between these higher pH values. The high value of antioxidant activity at pH 9 may be attributed to the improved activity of ionized species, despite the overall lower yield of phenolics and flavonoids at alkaline pH. The radical scavenging potential of DPPH radicals was significantly lower (indicated by a higher IC_50_ value) for extracts prepared in alkaline conditions, corroborating the observed decrease in total phenolic and flavonoid content at this pH. Conversely, the radical scavenging potential of UACPE against ABTS radical cations became increasingly potent as the pH rises, with the IC_50_ value for the alkaline extract being below 0.5 µL. This increased potency in stabilizing radical cations was likely due to the presence of conjugate base forms of phenolic acids and flavonoids in the extract, as the pKa of most hydroxyl, phenol, and carboxylic acid moieties is exceeded at pH 9.

**Figure 7:**
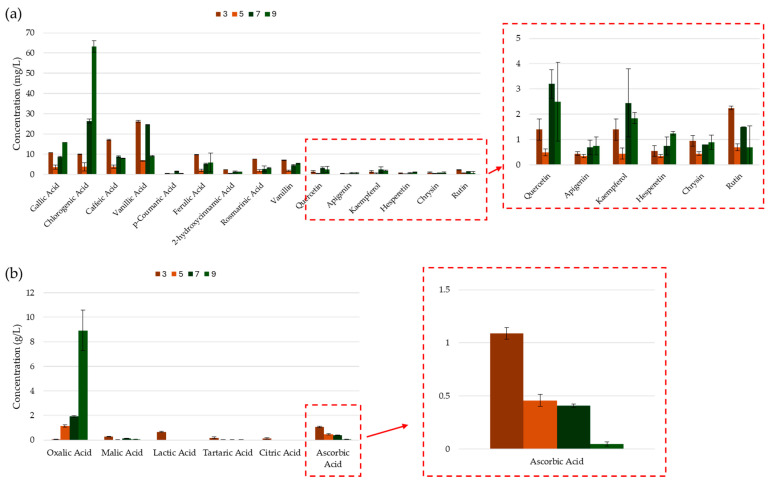
Concentrations of (**a**) phenolic compounds and (**b**) organic acids in the UACPE extracts prepared at various pH levels. Chromatograms for phenolic compounds and organic acids obtained through HPLC-UV are provided in the Supplementary Information.

A detailed analysis of individual phenolic compounds using HPLC revealed that the concentrations of gallic acid, chlorogenic acid (a derivative of caffeic acid and quinic acid), and hesperitin (flavanone aglycone of hesperidin) increased with elevated pH levels. In contrast, other phenolic acids, flavonoids, and their glycosides exhibited higher concentrations at either acidic pH (pH 3) or neutral pH (pH 7) (see Figure 7). Most of the phenolic acids and flavonoids tested in this study were extracted with the least efficiency at the slightly acidic pH of 5. This was not prominently highlighted in the chromometric assay results, suggesting the possible interference of co-extracted compounds (such as sugars or organic acids) with the assay’s reactions, or that the pH of the extract itself may have influenced the reaction and/or colorimetric response of the assay. The generally low mass transfer of phenolic compounds at pH 5 may be attributed to the ionization of an acidic moiety in the molecules (e.g., vanillic acid pKa_1_ = 4.51 [85]; caffeic acid pKa_1_ = 4.8 [86]). This ionization could lead to molecular fragmentation when subjected to ultrasonic energy bursts, unless sufficiently stabilized through charge delocalization and/or interactions with co-extracted compounds, such as proteins or organic acids. At higher pH levels (7 and 9), deprotonation of the less acidic moieties may occur. While this may render the ionized species more susceptible to degradation and/or oxidation, it could simultaneously enhance their solubility in the ethanol–buffer medium. Consequently, the balance between these antagonistic mechanisms will determine the recovery of phenolic compounds.

For example, the solubility of the flavonoid naringenin increased 314-fold from 9 × 10^−3^ ± 2 × 10^−3^ mM at pH 3.5 to 2.83 ± 0.18 mM at pH 8.5 [70]. This trend was also evident in the recovery of all organic acids, with the exception of oxalic acid, whose concentrations decreased substantially as the pH increased from 3 to 5 (see Figure 7). However, other phenomena may affect phenolic compounds like quercetin and rutin that have pKa_1_ values greater than 5. For instance, the ionic potential of the buffer–ethanol mixture may contribute to the denaturation of their storage sites within the plant material.

The observed increase in gallic acid and chlorogenic acid concentrations may be attributed to the hydrolysis of cellulosic materials, such as lignin and cellulose. Alkaline hydrolysis effectively disrupts the cellulose structure, facilitating the release of bound polyphenols. The substantial increase in recovered chlorogenic acid in its conjugate base form at pH 9 may explain the sharp drop in the IC_50_ value of ABTS radical cation scavenging. Similarly, hesperitin exhibits a steady increase in concentration with alkaline pH, likely due to the alkaline hydrolysis of its glycoside, hesperidin, which is abundantly present in orange peel [73]. In fact, although not quantified, the concentration of hesperidin in the alkaline extract appeared reduced when compared to neutral pH (see Figure S6 in Supplementary information).

### 2.3. Ultrasonic Parameter Settings

#### 2.3.1. Amplitude (%)

**Figure 8:**
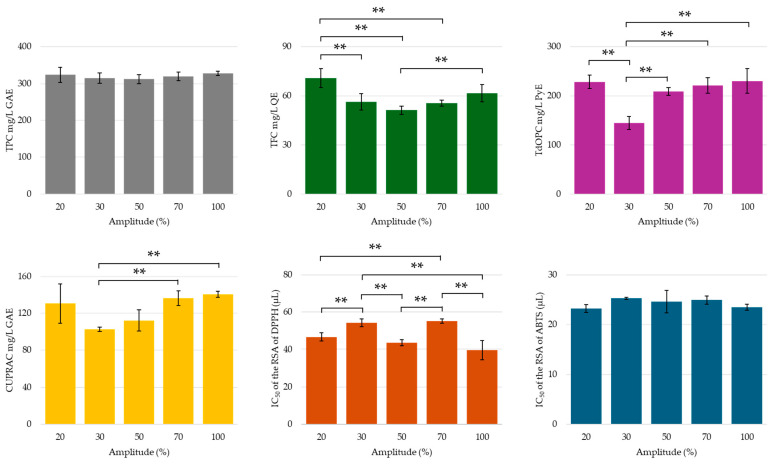
TPC, TFC, TdOPC, CUPRAC, and RSA of UACPE extracts prepared at various amplitudes of ultrasonic waves. Horizontal bars annotated with two asterisks indicate statistically significant differences between pairs with a 95% confidence level.

The amplitude of the ultrasonic waves is crucial for enhancing extraction efficiency; as the amplitude increases, the frequency of compression and rarefaction cycles also rises, leading to a greater release of compounds [32]. The results obtained from this study indicated that while there was no significant variation in total phenolic content with increasing amplitude, a U-shaped trend was observed for flavonoid and orthodiphenolic content, with the minimum points at 30 or 50% amplitude (see Figure 8). The lack of agreement between the TPC and other assays suggests that there may have been other influencing factors, for example, the reducing potency of co-extracted sugars, giving rise to non-representative results [87,88]. The CUPRAC antioxidant activity of the assays complemented the TFC and TdOPC trends, as the lowest activity was recorded for the extracts with lowest polyphenol content (30% amplitude). Changes in amplitude did not significantly affect the ABTS radical cation scavenging potency of the extracts and no distinctive trend was observed for DPPH RSA.

**Figure 9:**
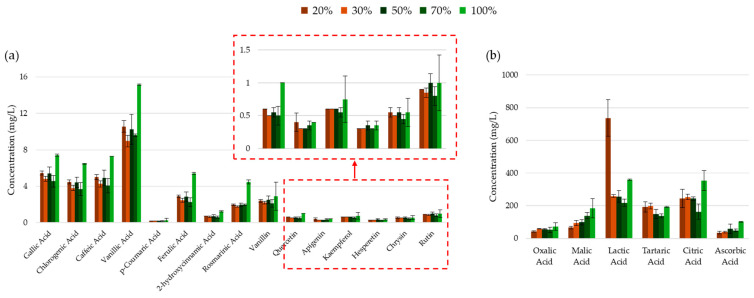
Concentrations of (**a**) phenolic compounds and (**b**) organic acids obtained though HPLC-UV separation and analysis of the UACPE extracts prepared with various amplitudes.

A more in-depth analysis of the individual polyphenols by HPLC-UV provides insights into the non-conclusive results observed in the chromometric assays. The results collected in Figure 9a show that, as the amplitude increased from 20 to 70%, only slight changes were observed in the concentrations of the recovered compounds. However, a notable increase in the yield of most phenolic compounds, particularly hydroxybenzoic and hydroxycinnamic acids, was observed at 100% amplitude. These results suggest that, as anticipated, increasing the US power output improved the overall efficacy of the extraction [32,89,90,91]. In general, the organic acid recovery improved steadily with the increase in amplitude, with the exception of lactic acid, whose optimal recovery occurred at the lowest amplitude setting of 20%. This exceptionally high concentration of lactic acid may have influenced the chromometric assay results, explaining the disproportionately high values observed at 20% amplitude [32,89,90,91].

#### 2.3.2. Pulse (%)

**Figure 10:**
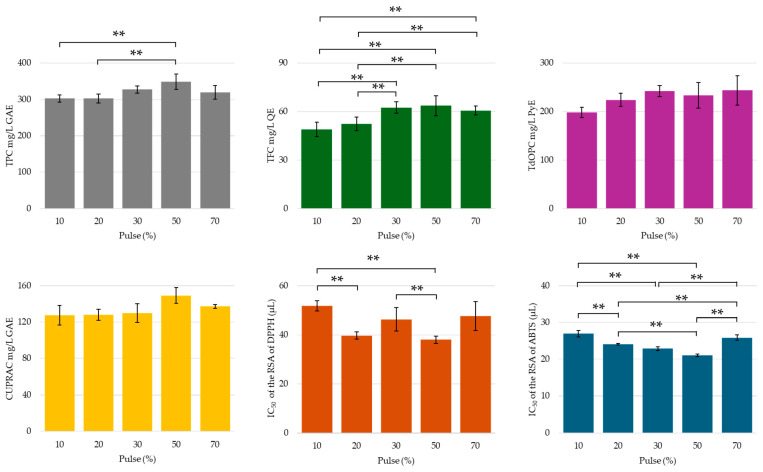
TPC, TFC, TdOPC, CUPRAC, and RSA of UACPE extracts prepared at various pulses of ultrasonic waves. Horizontal bars annotated with two asterisks indicate statistically significant differences between pairs with a 95% confidence level.

In this study, pulses of 10, 20, 30, 50, and 70% at an amplitude of 50% were investigated. Higher pulse rates were avoided, due to the splatter and loss of peel and extract solution from the jacketed flask. The chromometric assay results show a steady increase in phenolic and flavonoid content up to a pulse of 50%, followed by a slight decrease at a pulse of 70% (see Figure 10). Although higher pulse percentages were anticipated to enhance the extraction of phenolic compounds, the observed plateau and decline may be attributed to an extended US energy transmission, potentially leading to the degradation and/or oxidation of these compounds, despite constant cooling during the extraction process [20,24,27,36,40]. For context, a pulse of 10% delivered an energy output of 1960 Ws, a pulse of 30% delivered 6859 Ws, and a pulse of 70% delivered 14,252,400 Ws. Orthodiphenolic content steadily increased up to 30% pulse, followed by a plateau, suggesting that the OH moieties in these molecules stabilized ionized and/or oxidized forms. Similarly, the CUPRAC activity mirrored the trends in phenolic and flavonoid yields, as the presence of these compounds enhanced the antioxidant activity of the extract. The IC50 values for ABTS radical cation scavenging followed a similar trend, with the maximum activity (lowest IC50) observed at 50% pulse, followed by a decrease at 70% pulse. The higher US energy achieved through increased pulsing frequency likely contributed to the ionization of phenolic compounds into their conjugate base forms, thus improving the stabilization of radical cations. However, at 70% pulse, the excessive energy appeared to fragment and degrade the ionized compounds, diminishing this effect.

**Figure 11:**
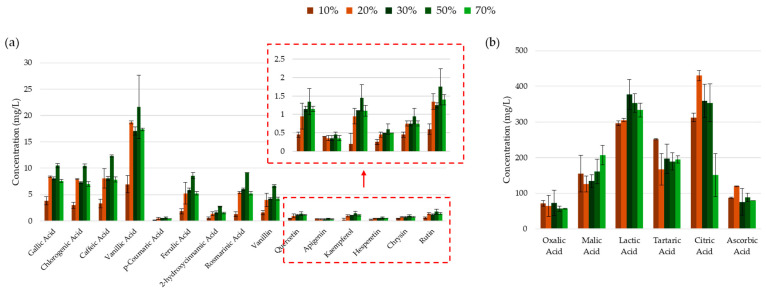
Concentrations of (**a**) phenolic compounds and (**b**) organic acids obtained though HPLC-UV separation and analysis of the UACPE extracts prepared with various pulse settings.

The HPLC profile assessment of UACPE extracts indicated an increase in the extracted concentration of most phenolic acids, flavonoids, and their glycosides from a pulse of 10% to 50% (see Figure 11). This was followed by a decline at a pulse of 70%, further emphasizing the degradation of phenolic compounds as the ultrasonic power was increased. These findings are generally in agreement with the assay results. The sharp increase in the highly ionizable hydroxybenzoic and hydroxycinnamic acids at 50% pulse corroborates the lowest IC_50_ value for ABTS radical cation scavenging. Interestingly, the organic acids showed a similar trend; however, unlike the phenolic compounds, their peak concentration was in general achieved using a pulse of 30% or lower (see Figure 11). For example, pulse settings greater than 30% were observed as diminishing the recovery of lactic acid, most likely due to fragmentation of its conjugate base. This highlights that the generally lower pKa_1_ values of these organic acids made them increasingly susceptible to ionization and degradation by lower energy US pulses.

#### 2.3.3. Probe Size and Immersion Depth

**Figure 12:**
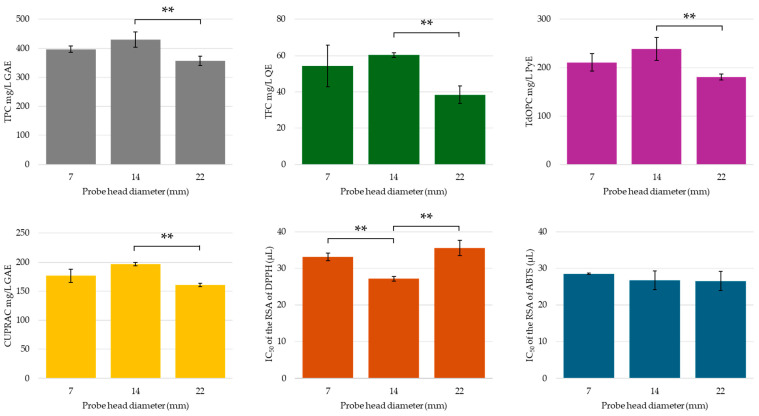
TPC, TFC, TdOPC, CUPRAC, and RSA of UACPE extracts prepared at various probe head diameters. Horizontal bars annotated with two asterisks indicate statistically significant differences between pairs with a 95% confidence level.

Probe factors like position, depth, and diameter affect extraction yield, with larger probes generally expected to deliver a higher intensity and enhance the efficiency by disrupting cellular structures [35,36,37,40]. In this investigation, no significant differences in total phenolic, flavonoid, or orthodiphenolic content, nor antioxidant activity, were observed between the smallest probe size (7 mm) and the 14 mm probe (see Figure 12). This aligns with the findings by Silva and Saldaña, who reported that probe diameters did not influence the extraction yields in their study [37]. However, in the case of this study, slight (although not statistically significant) increases in flavonoids and orthodiphenolics were associated with a significantly lower IC50 value for DPPH radical scavenging activity in the extract prepared with the 14 mm probe. In contrast, significant reductions in all assay results (except for ABTS RSA) were observed when comparing the 14 mm and 22 mm probes. This reduction was also evident in the HPLC analysis of phenolic compounds and organic acids (see Figure S5). The overall decrease in phenolic compounds yield, antioxidant activity, and radical scavenging activity with the 22 mm probe may be attributed to the vigorous agitation of the extraction medium, leading to material losses and adherence to the sides of the flask.

**Figure 13:**
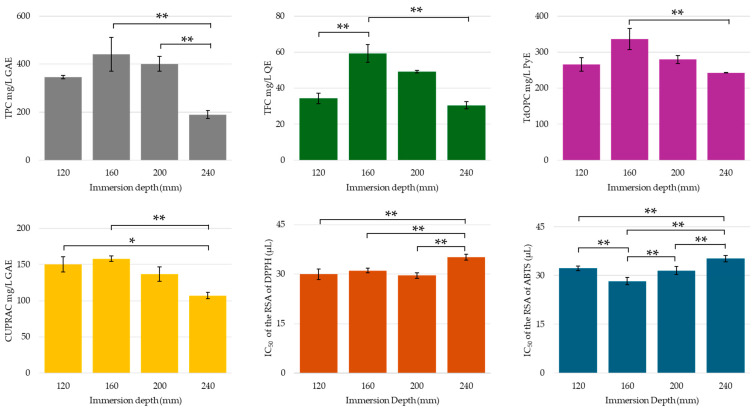
TPC, TFC, TdOPC, CUPRAC, and RSA of UACPE extracts prepared at various probe immersion depths. Horizontal bars annotated with two asterisks indicate statistically significant differences between pairs with a 95% confidence level. Horizontal bars annotated with one asterisk indicate statistically significant differences between pairs with a 90% confidence level.

In the case of probe immersion depth, a U-shaped trend was obtained for the total phenolic, flavonoid, and orthodiphenolic contents, with maximal values recorded with an immersion depth of 160 mm (see Figure 13). The phenolic compound content was found to be significantly different from that obtained using a 240 mm depth, i.e., a depth at which the probe was in direct physical contact with the dried orange peel. The low recovery of phenolics and flavonoids with the 240 mm immersion depth was also reflected in the CUPRAC antioxidant activity, DPPH radical scavenging activity, and ABTS radical cation scavenging activity of the extract. In fact, the DPPH RSA of the extract prepared with the largest immersion depth was significantly lower than those of the other extracts. In terms of ABTS radical cation scavenging, the extract obtained with a 160 mm immersion depth attained the lowest IC_50_ value, complementing the findings that the flavonoid and orthodiphenolic yield was greatest under this condition. It is highly likely that the observed reduction in the yield of polyphenolic compounds, antioxidant activity, and RSA was a result of the greater agitation of the solution with the higher immersion depths, which led to losses of peel and solvent, as well as greater US energy transfer, which may have caused degradation of the phenolic compounds. For these parameters, the concentrations of phenolic compounds and organic acids obtained through HPLC-UV are provided in the Supplementary Information (Figure S9).

### 2.4. Extraction Time

**Figure 14:**
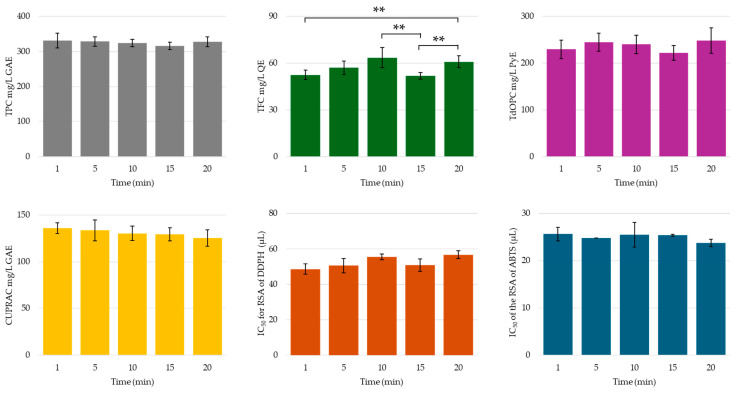
TPC, TFC, TdOPC, CUPRAC, and RSA of UACPE extracts prepared with various extraction times. Horizontal bars annotated with two asterisks indicate statistically significant differences between pairs with a 95% confidence level.

Longer extraction times are generally associated with improved yields of phenolic compounds [32,33,34,59]; however, there are reported instances in which prolonged extraction times did not significantly improve the yield of bioactive compounds, since saturation was reached abruptly [32]. In another work, de Miera et al. [40] observed that there was a substantial decrease in hesperidin content with prolonged extraction times, and the authors argued that this was most likely due to degradation of this glycoside when temperatures rose to c. 333 K during extraction. In this investigation, the extraction time was only found to significantly affect the total flavonoid content in a U-shaped manner, with the greatest recovery of flavonoids obtained after 10 min of sonication and the subsequent reduction in yield likely due to degradation (see Figure 14). The lack of effect of the extraction time on the phenolic content, antioxidant activity, and radical scavenging activity highlights the great efficacy of probe-coupled UAE, since 1 min was sufficient for polyphenol saturation.

**Figure 15:**
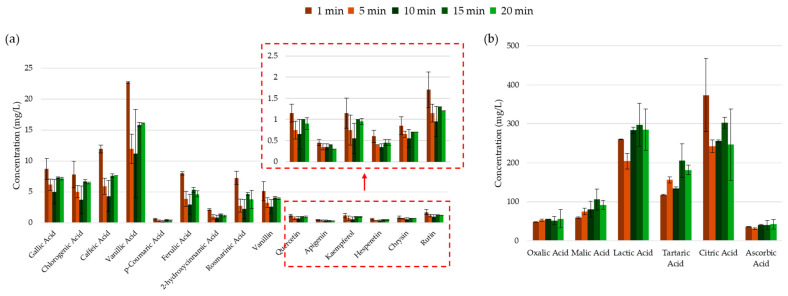
Concentrations of (**a**) phenolic compounds and (**b**) organic acids obtained though HPLC-UV separation and analysis of the UACPE extracts prepared with various extraction times.

A chemoinformatic approach revealed detailed differences between the extracts that were not observable through colorimetric assays. As shown in Figure 15a, the maximum concentration of each identified and quantified phenolic acid and flavonoid was achieved after just 1 min of UAE. This finding does not align with the TFC assay results, suggesting that the complexation assay may have been influenced by the presence of high amounts of citrate and ascorbate. Prolonged extraction times (5 to 10 min) led to the degradation of both phenolic acids and flavonoids, likely due to the ionization and fragmentation caused by extended exposure to US energy. However, yields improved again at 15 to 20 min, although they remained lower than those obtained after just 1 min of extraction. This suggests that beyond 10 min, the rate of extraction surpasses the rate of degradation. Nevertheless, the yield after 20 min does not match the yield achieved after 1 min, further highlighting the efficacy of UAE. A similar trend was observed for citric acid, suggesting that citrate ion may potentially stabilize extracted polyphenols. However, once the citrate ions started to fragment, the polyphenols appeared to degrade as well. Moreover, the improved recovery of the simpler organic acids, such as malic, lactic, and tartartic acids, over prolonged extraction times was likely due to the breakdown of citric acid and hydroxycinnamic acid branches under extended US exposure.

### 2.5. Summary and Perspective

This investigation showed that optimized yields may be obtained by lowering the solid-to-solvent ratio, in order to improve mass transfer dynamics. Whilst lowering this ratio can maximize extraction efficiency, the use of excessive solvent can lead to an increase in costs associated with subsequent operations, such as the concentration and filtration of the extracts. Use of large volumes of solvent would also result in the generation of greater volumes of waste. One must also consider that if the extracts will be used to functionalize food products, the ethanol solvent must be removed. Particle size also played a critical role, with the optimal range identified as 710–45 µm. Larger and smaller sizes resulted in extracts with lower polyphenol contents and antioxidant activity. However, on an industrial scale, separating smaller particle sizes may not be economically feasible.

The solvent composition, particularly ethanol concentration and pH, emerged as influential variables in the UAE from orange peel. Although phenolic acids and flavonoids generally have improved solubility at higher ethanol concentrations, the optimal yields, antioxidant activity, and radical scavenging activity were achieved in the range of 10 to 25% ethanol. Chemometric analysis suggested that co-extraction of organic acids, particularly citric acid, in aqeous ethanol media contributed to these results, due to stabilizing effects. With regards to pH, a neutral pH of 7 yielded the optimal phenolic compound recovery, with negligible organic acids except oxalic acid. Alkaline pH facilitated cell wall hydrolysis, releasing significant amounts of ionized gallic and chlorogenic acids, which contributed to the observed differences in activity.

Four variables related to ultrasonic power were investigated, showing that maximization of pulse, probe head diameter, and immersion depth often did not result in improvements in phenolic compound yields, antioxidant activity, or radical scavenging activity. Through chemometric analysis, it was shown that the highest settings of pulse, probe head diameter, and immersion depth resulted in lower compound recovery, which may have been due to splattering of peel from the extraction medium or compound degradation caused by temperature, ionization, or fragmentation. The exception within this sub-category was the amplitude setting, whereby chemometric analysis showed that maximization of amplitude (up to 100%) aided in the recovery of higher concentrations of phenolic acids, flavonoids, and organic acids, something that was not apparent with the chromometric assays alone. These findings highlight the importance of distinguishing between amplitude and pulse effects on compound stability. Even though an increase in both parameters results in higher US power and energy output, under the conditions tested in this study, only maximization of amplitude improved the recovery of phenolic compounds from the dried orange peel, whilst care must be taken to optimize the pulse parameter to avoid undesirable degradation of the extracted polyphenols.

Extraction time was not found to significantly affect the antioxidant activity and radical scavenging activity of the extracts. However, through chemometrics, it was observed that the maximum concentration of phenolic acids and flavonoids was achieved with one minute of extraction time, underscoring the efficacy of probe-coupled ultrasonic extractions. Prolonging the extraction time to 10 min resulted in degradation of the phenolic compounds in the extract; however, further prolongation to 15 and 20 min resulted in improved recovery, likely due to an improved mass transfer overcoming the rate of degradation. In this context, it would be interesting to investigate the coupled effects of extraction time and the extraction medium’s polarity and ionic potential, since the latter two factors were found to significantly influence the stabilization of extracted phenolic compounds, likely through the co-extraction of organic acid conjugate bases (such as citrate and ascorbate).

Taken together, these findings fulfill the aims of this study by providing a comprehensive screening of nine specific extraction parameters and highlighting the conditions (or their ranges) that favor maximizing the extraction of key classes of compounds. For instance, the results presented here indicate that low pH, short extraction times, 10 to 25% ethanol, moderate pulses, and high amplitude favored phenolic acid and flavonoid recovery, as well as antioxidant activity. Interestingly, it was also found that alkaline conditions specifically enhanced the chlorogenic acid content.

Before concluding, it is important to note the limitations of this one-factor-at-a-time approach. Although numerous experiments were conducted to assess all nine variables individually, no information on the combined effects of variables could be collected. Future studies employing response surface methodologies (RSM) could elucidate these combined effects. Additionally, quantification of sugars, proteins, and fibers was not included. Thus, the effects, if any, of the co-extraction of these classes of compounds on the chemometric and chromometric results could not be highlighted. Furthermore, certain chromometric assay results were non-conclusive due to the non-specific nature of the assay reactions, something which was alleviated by HPLC analysis. It was also beyond the scope of this work to identify and quantify all elution peaks in the chromatogram pertaining to the phenolic compounds.

The results obtained show that ultrasonic-assisted extraction may be deemed a highly efficient method to valorize orange peel waste if coupled with the optimal operational range of each of the chemical and physical variables. One method to valorize this waste is in the context of the functionalization of food products with added nutritional value or prolonged product stability. The results obtained in this study may serve as a guide for targeting the extraction of compound classes or even specific individual compounds. For instance, citrus fruit flavones are associated with reducing neurodegenerative diseases, cardiovascular disease, and type 2 diabetes [9,13,92,93,94], and in this study, low pH, short extraction times, 10 to 25% ethanol, moderate pulses, and high amplitude were identified as effective conditions for this class of flavonoids. Similarly, low pHs or 25% ethanol was found to be optimal for the extraction of ascorbic acid, a vitamin with various health benefits that is not biochemically available in humans and which can also act as an antioxidant to extend product shelf-life [95,96].

On a different note, extraction at alkaline pH readily increased the chlorogenic acid content in the extract, a compound that is linked with a wide range of health benefits, such as cardioprotection, neuroprotection, and decreased blood pressure [97,98,99]. However, for functionalization of food products, the use of such alkaline extracts would require lowering the pH and to removing the high levels of anti-nutritional compounds like oxalic acid and oxalate present in the crude extract [100]. Looking forward, an important future perspective consists of conducting more focused studies to evaluate the most influential variables through multi-response technique, using similar responses and analysis methods as justified in this work. Such studies would help define an optimized extraction parameter profile to improve phenolic compound recovery, antioxidant activity, and radical scavenging activity, thereby maximizing the efficacy of the UAE technique for food waste valorization.

## 3. Materials and Methods

### 3.1. Plant Material

Citrus peel derived from freshly juiced Navelina oranges (*Citrus sinensis*), procured from Mgarr Farms (Mgarr, Malta) and Koperattivi Malta (Qormi, Malta), was processed for further use. The peel, comprising a mixture of albedo, flavedo, and segment walls, was coarsely chopped and subjected to drying under convection at a temperature of 65 °C for a duration of 3 to 4 days, or until the moisture content fell below 5%. While it is acknowledged that drying temperature can affect the concentration of natural compounds present within the peel, this parameter was kept constant throughout the study. Subsequently, the dried plant material was pulverized using a standard blender and stored in a vacuum desiccator, shielded from light, until required for application. Particle size fractions were obtained via a Retsch AS 200 shaker (Haan, Germany) and appropriate stainless-steel mesh sieves.

### 3.2. Chemicals

The following compounds were procured from Sigma-Aldrich (St. Louis, MO, USA): Folin–Ciocalteu reagent, 2,2-diphenyl-1-picrylhydrazyl (DPPH), and 2,2′-azino-bis(3-ethylbenzothiazoline-6-sulfonic acid) diammonium salt (ABTS), along with gallic acid, protocatechuic acid, 4-hydroxybenzoic acid, vanillic acid, chlorogenic acid, *p*-coumaric acid, 2-hydroxycinnamic acid, vanillin, ferulic acid, caffeic acid, rosmarinic acid, quercetin, apigenin, rutin, kaempferol, hesperetin, hesperidin, and 5,7-dihydroxyflavone. Additionally, potassium persulfate was also sourced from the same supplier. Sodium bicarbonate, sodium hydroxide, aluminum chloride, sodium molybdate dihydrate, and sodium nitrite were obtained from Biochem Chemopharma (Cosne-Cours-sur-Loire, France), while ethanol, copper chloride, methanol, and acetonitrile were acquired from VWR (Rosny-sous-Bois, France).

### 3.3. Preparation of Ultrasonic Assisted Citrus Peel Extract (UACPE)

The citrus peel was ground and sieved before being weighed and transferred to a 100 mL jacketed media bottle. Then, 50 mL of solvent with varying concentrations of ethanol was added. Ultrasonic extraction was performed using a direct probe immersed in the solution with a 400 W, 24 kHz UP400St ultrasonic processor (Hielscher Ultrasonics GmbH, Teltow, Germany). The temperature of the extraction medium was kept at a constant 25 °C by means of a refrigerated circulating bath (Witeg WCR-P12, Wertheim, Germany), moving water through the media bottle’s jacket. Following extraction, the contents of the jacketed media bottle were transferred to a 50 mL falcon tube and underwent centrifugation at 4000 rpm for 10 min at 5 °C. The resulting supernatant and residue were then subjected to further analysis. With the aid of a vacuum pump, the supernatant layer was filtered using Whatman Grade 1 filter paper (Sigma-Aldrich, St. Louis, MO, USA) and stored in a brown flask at −20 °C until analysis. This study assessed the impact of various extraction variables, including time (1, 5, 10, 15, and 20 min), solid-to-solvent ratio (1:10, 1:20, 1:40, 1:50, 1:60, and 1:100 g/mL), particle size (≤45, 90–45, 125–90, 180–125, 250–180, 355–250, 710–355, and 1400–710 μm), pulse (10, 20, 30, 50, 70%), amplitude (20, 30, 50, 70, and 100%), ethanol concentration (0, 10, 25, 50, and 75%), pH (3, 5, 7, and 9), ultrasonic probe head diameter (7, 14, and 22 mm), and depth of probe immersed in the solution (120, 160, and 200 mm), using the one-factor-at-a-time statistical method described in Table 1.

### 3.4. Colourimetric Assays

#### 3.4.1. Determination of Total Phenolic Content (TPC)

The determination of TPC in the UACPE extracts was conducted using the Folin–Ciocalteu colorimetric method as described by Singleton et al. [101]. A calibration curve was established with gallic acid (Sigma-Aldrich, Saint Louis, MO, USA), yielding an R^2^ value of 0.97 and the equation y = 0.003x + 0.0547. In this assay, 20 μL of the extract was subjected to oxidation with 100 μL of a 5-fold diluted Folin–Ciocalteu reagent. The reaction was subsequently neutralized by the addition of 80 μL of 7.5% Na_2_CO_3_. The microtiter plate was then incubated in the dark at room temperature for two hours, after which the absorbance was measured at 630 nm using a microtiter plate reader (SPECTROstar Nano BMG, Ortenberg, Germany). In the case of solid:solvent ratios of 1:10 and 1:20 g/mL, the extract was diluted by a factor of 10, such that absorbances were reduced below a value of 2.

#### 3.4.2. Determination of Total Flavonoid Content (TFC)

The total flavonoid content was determined using the method described by Mabry, Markham, and Thomas, with slight adjustments [102]. In this procedure, 25 μL of extract was combined with 10 μL of 10% aluminum chloride, 10 μL of 7% *w*/*v* sodium nitrite, and 80 μL of distilled water. The microtiter plate was then left at room temperature for 30 min, followed by the addition of 100 μL of 1 M NaOH solution. After vigorous shaking, the absorbance for the reaction was measured at 450 nm. A calibration curve was established using quercetin with an R^2^ value of 0.97 and a regression equation of y = 0.00297x + 0.1054.

#### 3.4.3. Determination of Total Ortho-Diphenolic Content (TdOPC)

The concentrated extracts were analyzed for total ortho-diphenolic content using a modified Arnow colorimetric method based on established protocols [103,104]. A calibration curve was prepared using protocatechuic acid as the standard, resulting in a regression equation of y = 0.00125x + 0.0539 and an R^2^ value of 0.96. For each measurement, 20 μL of the extract was transferred to a 96-well microtiter plate and mixed with 20 μL of 1 M HCl. Subsequently, 20 μL of freshly prepared Arnow’s reagent, consisting of sodium molybdate dihydrate (10 g) and sodium nitrite (10 g) dissolved in 100 mL of a 1:1 ethanol-water mixture, was added. After vigorous shaking and a 15-min reaction time, 80 μL of water and 40 μL of 1 M NaOH were introduced into the mixture. The absorbance of the resulting solution was measured at 405 nm using a microtiter plate reader.

#### 3.4.4. Determination of Cupric Reducing Antioxidant Power Assay (CUPRAC)

The antioxidant capacity of the extracts, based on their ability to reduce cupric ions (CUPRAC), was measured using a modified version of the method developed by Apak et al. [105]. A standard calibration curve was prepared with gallic acid (Sigma-Aldrich), resulting in a regression equation of y = 0.0057x − 0.1783 and an R^2^ value of 0.97. For the assay, 20 μL of the extract was mixed with 100 μL of a 10 mM CuCl_2_ solution. Next, 100 μL of 1 M ammonium acetate buffer at pH 7.0 and 100 μL of a 7.5 mM neocuproine solution in ethanol were added sequentially. The reaction mixture was left to incubate at room temperature for 30 min, after which the absorbance was measured at 405 nm using a microplate reader (SPECTROstar Nano BMG, Ortenberg, Germany).

#### 3.4.5. Determination of DPPH Radical Scavenging Activity (RSA)

The DPPH RSA of the extracts was assessed utilizing the methodology outlined by Rahman et al. [16], with slight modifications. To evaluate the RSA of phenolic compounds extracted from UACPE, daily preparation of a 60 μM DPPH stock solution in methanol was conducted, which was subsequently stored in the dark at 4 °C. A volume of 50 μL of the UACPE was introduced into the first well, and 25 μL of methanol was added to the five adjacent wells in a 96-well microtiter plate. A serial two-fold dilution of the UACPE stock was conducted to construct a response curve against volume of UACPE. Additionally, a series of negative DPPH controls were established within the same plate by adding 50 μL of methanol to each well. Subsequently, 150 μL of the methanolic DPPH solution was added to each well, and the reaction was allowed to proceed for 30 min in the dark. The absorbance was then recorded at 560 nm using a microtiter plate reader. IC_50_ values, i.e., the volume of UACPE extract required to drop the RSA to 50%, were calculated using linear or logarithmic regression, depending on the r^2^ of fit of the response curve, using MS Excel Software.

#### 3.4.6. Determination of ABTS Radical Cation Scavenging Activity (RSA)

The ABTS RSA of the extracts was assessed according to the method described by Rajurkar and Hande [106]. The ABTS radical cation was generated by combining a 7 mM stock solution of ABTS with 2.45 mM of potassium persulfate and allowing the mixture to stand in the dark at room temperature for 12 h. The concentration of the resulting blue–green ABTS radical solution was adjusted to an absorbance of 0.700 at 734 nm using methanol. Subsequently, 40 μL of the UACPE was added to a well and further diluted through a serial two-fold dilution in a 96-well microtiter plate. Negative ABTS controls were included in the same plate by adding 40 μL of methanol to each well. Following this, 280 μL of ABTS radical solution was added to each well, and the reaction was incubated for 5 min at 30 °C. The absorbance was then measured at 450 nm using a microtiter plate reader. IC_50_ values were calculated using the method described in Section 3.4.5.

### 3.5. Separation and Determination of Compounds in UACPE Using HPLC-UV

#### 3.5.1. Separation of Phenolic Compounds

The HPLC system (Shimadzu LC-20 AB, Kyoto, Japan) was configured with a binary pump, an autosampler (SIL-20AC), and a UV/Vis detector (SPD-20AV) operating at 280 nm and 320 nm. A 10 μL injection volume was analyzed on an ACE^®^ C18 analytical column (250 × 4.6 mm, 5 μm particle size; Aberdeen, UK). The mobile phase consisted of (A) 0.2% trifluoroacetic acid in water and (B) a methanol/acetonitrile mixture, delivered at a flow rate of 1.2 mL/min. The gradient elution program began with 80% (A)/20% (B) from 0 to 10 min, shifted to 70% (A)/30% (B) from 10 to 20 min, then to 50% (A)/50% (B) from 20 to 25 min, followed by 25% (A)/75% (B) from 25 to 30 min, and 100% (B) from 30 to 31 min. A re-equilibration step at 80% (A)/20% (B) was included for the final 2 min. The column temperature was precisely maintained at 45 °C using a Shimadzu CTO-10AC oven, while the sample chamber was cooled to 4 °C to prevent sample degradation. The detector acquisition time and total run duration were set to 34 min.

All standards and samples were prepared and filtered through 0.45 µm PVDF syringe filters before analysis. Standards, with purities ranging from 85.18% to 99.99%, were quantified using a six-point calibration curve spanning concentrations of 0.1 to 100 µg/mL. Phenolic compounds were identified based on their retention times (RT), and quantification followed. System suitability was verified through six consecutive injections of syringic acid at a concentration of 50 µg/mL, with an acceptance criterion of a relative standard deviation (RSD) below 5%. Intermediate precision was assessed by injecting syringic acid at the same concentration after every 10 sample injections, with measurements performed in duplicate. Quantification parameters included evaluations of linearity, recovery, limit of quantification (LOQ), and limit of detection (LOD), determined using externally prepared calibration curves for individual compounds.

#### 3.5.2. Separation of Organic Acids

The HPLC analysis was carried out using a Shimadzu LC-20 AB system (Kyoto, Japan), which was equipped with a binary pump, an autosampler (SIL-20AC), and a UV/Vis detector (SPD-20AV), set to monitor absorbance at 210 nm and 250 nm. A 10 μL injection volume was employed for separation on an ACE^®^ C18 analytical column (250 × 4.6 mm i.d., 5 μm particle size; Aberdeen, Scotland). Separation was achieved through isocratic elution using a pH 2.5 phosphate buffer as the mobile phase, with a constant flow rate of 1.2 mL/min. The column temperature was controlled at 45 °C using a Shimadzu CTO-10AC column oven, with a tolerance limit of 40 °C, while the sample chamber was maintained at 4 °C to prevent degradation of the samples. The total analysis time was 10 min, with the detector data acquisition set for this duration.

All standards and samples were initially prepared and filtered through PVDF 0.45 µm syringe filters. Alongside the standards (purity between 85.18% and 99.99%, the compound quantification, following organic acid identification through RT, utilized a 6-point calibration curve ranging from 0.1 to 10 g/L. System suitability was verified through six consecutive injections of citric acid at a concentration of 5 g/mL, with an acceptance criterion of relative standard deviation (RSD) below 5%. Intermediate precision was evaluated by injecting citric acid at the same concentration after every 10 sample injections, measured in duplicates. The quantification involved determining the linearity, recovery, LOQ, and LOD of individual compounds using externally prepared calibration curves.

### 3.6. Univariate Statistical Analysis

All tests were performed in triplicates, and the means ± SD (M ± SD) of the data were reported. An analysis of variance (ANOVA) test was used to evaluate the presence of significant differences at 95% and 90% confidence level. For this purpose, statistical analysis was accomplished using IBM SPSS V22 and JMP software (version number: 10.0.0), drawing the related charts using MS Excel 2023 software. The horizontal lines in the bar chart plots of the data from the chromometric assays indicate statistically significant differences between experimental trials, with double asterisks annotating differences with a 95% confidence level and double asterisks annotating differences with a 90% confidence level.

## 4. Conclusions

The present study comprehensively evaluated nine variables in the UAE of bioactive compounds from dried orange peel. Key findings reveal that the solvent composition, especially ethanol concentration and pH, were the most influential factors in the variation in polyphenol yield and co-extraction of phenolic compounds and organic acids, namely, citric acid. Ideal ethanol concentrations were identified between 10 and 25%; higher concentrations, unexpectedly, reduced the yields of identified polyphenols, suggesting additional solubility-limiting factors may have been at play. Alkaline conditions (pH 9) enhanced the radical cation scavenging potential, although containing a decreased yield of the phenolic compounds, whereas acidic (pH 3) and neutral (pH 7) conditions were ideal for extracting phenolic acids and flavonoids. Solid parameters, including the solid-to-solvent ratio and particle size, often overlooked in the literature pertaining to UAE from orange peel, also significantly impacted the extraction efficiency. Lower solid-to-solvent ratios of 1:60 and 1:100 facilitated mass transfer and improved yields, while particle size optimization—excluding the largest (≥710 µm) and smallest (≤45 µm) ranges—enhanced the recovery and antioxidant activity. In terms of the ultrasonic power parameter settings, the maximum amplitude enhanced the recovery of phenolic acids and flavonoids, though pulse limitations up to 50% were necessary to prevent degradation of phenolic compounds and co-extracted organic acids. Additionally, the medium-sized probe head and mid-level immersion improved the extraction efficiency, likely due to reducing splattering and content loss from the extraction medium. Interestingly, extraction time was not a critical factor, with the peak polyphenol yield achieved after just one minute, thus highlighting the rapid efficacy of probe-coupled UAE. Extended extraction led to decreased yields, likely due to compound ionization and fragmentation under prolonged exposure to ultrasonic energy. This study served as an initial exploration into the operational limits of non-thermal UAE for orange peel extraction, especially for those physical and chemical variables which have been overlooked in previous multi-response optimization studies in the literature. Future work will entail the implementation of the effects, boundaries, and ranges learned here in optimization studies concerning the valorization of orange peel waste using this green technology. The findings presented in this work underscore the potential of UAE as a highly efficient method for valorizing citrus peel waste on an industrial-scale in high-throughput applications within the food processing industry.

## Figures and Tables

**Table 1:** One factor-at-a-time experimental design.

Variable	A	B	C	D	E	F	G	H	I
Solid:Solvent Ratio (*w*/*v*, g/mL)	1:10	1:50							
	1:20								
	1:40								
	1:50								
	1:60								
	1:100								
Time (min)	10	1	10						
		5							
		10							
		15							
		20							
Amplitude (%)	50		20	50					
			30						
			50						
			70						
			100						
Pulse (%)	20			10	20				
				20					
				30					
				50					
				70					
Ethanol (*v*/*v*, %)	50				0	50			
					10				
					25				
					50				
					75				
pH	No buffer					3	No buffer		
						5			
						7			
						9			
Particle size (µm)	<710						1400–710	<710	
							710–355		
							355–250		
							250–180		
							180–125		
							125–90		
							90–45		
							<45		
Probe head diameter (mm)	7							7	7
								14	
								22	
Probe Immersion depth (mm)	120								120
									160
									200

## Data Availability

Any additional data not presented in the article and Supplementary Materials are available from the authors on request.

## Supplementary Materials

The following supporting information can be downloaded at: https://www.mdpi.com/article/10.3390/molecules30030648/s1, Table S1: External Standard Calibration Curves of the quantified Phenolic Acids and Flavonoids; Table S2: LOD and LOQ for the chromometric assays; Figure S1: Calibration Curve for the determination of Total Phenolic Content (TPC); Figure S2: Calibration Curve for the determination of Total Flavonoid Content (TFC); Figure S3: Calibration Curve for the determination of Total Orthodiphenolic Content (TdOPC); Figure S4: Calibration Curve for the determination of CUPRAC Antioxidant activity; Figure S5: Example of chromatograms with variation in Ethanol concentration: (a) full-scale and (b) zoomed-in HPLC-UV chromatograms of the phenolic compounds in UACPE extracts with various ethanol concentrations. Annotations: (1) Gallic acid, (2) Chlorogenic acid, (3) Caffeic acid (4) Vanillic acid, (5) p-Coumaric acid, (6) Vanillin, (7) Ferulic acid, (8) 2-hydroxyccynnamic acid, (9) Rosmarinic acid, (10) Hesperidin, (11) Quercetin, (12) Apigenin, (13) Kaempferol, (14) Hesperetin, (15) Chrysin, (16) Rutin; Figure S6: Example of chromatograms with variation in pH of extraction medium: (a) full-scale and (b) zoomed-in HPLC-UV chromatograms of the phenolic compounds in UACPE extracts with various ethanol concentrations. Annotations: (1) Gallic acid, (2) Chlorogenic acid, (3) Caffeic acid (4) Vanillic acid, (5) p-Coumaric acid, (6) Vanillin, (7) Ferulic acid, (8) 2-hydroxyccynnamic acid, (9) Rosmarinic acid, (10) Hesperidin, (11) Quercetin, (12) Apigenin, (13) Kaempferol, (14) Hesperetin, (15) Chrysin, (16) Rutin; Figure S7: Effect of solid (peel) to liquid (solvent) ratio on the concentrations of phenolic compounds and organic acids (legend units = g/mL); Figure S8: Effect of particle size on the concentrations of phenolic compounds and organic acids (legend units = µm); Figure S9: Effect of probe size diameter on the concentrations of phenolic compounds and organic acids (legend units = mm); Figure S10: Effect of probe immersion depths on the concentrations of phenolic compounds and organic acids (legend units = mm).
