# Supplementary material for: A Comprehensive Analysis of Non-Thermal Ultrasonic-Assisted Extraction of Bioactive Compounds from Citrus Peel Waste Through a One-Factor-at-a-Time Approach
Source: Molecules. 2025 Feb 1;30(3):648. doi: 10.3390/molecules30030648 (PMC11820553; doi:10.3390/molecules30030648)
Supplement: Supplementary file 1 [file molecules-30-00648-s001.zip › molecules-3428156-supplementary.pdf]

Table S1: External Standard Calibration Curves of the quantified Phenolic Acids and Flavonoids

Standard	Slope	Intercept	Standard Error	LOD (mg/L)	LOQ (mg/L)	R^2	%Accuracy
Gallic Acid	13181	63	832	0.266	0.807	0.999	98.06
Chlorogenic Acid	6680	-889	704	0.505	1.531	0.998	97.59
Caffeic Acid	14749	505	1848	0.600	1.819	0.998	104.55
Vanillic Acid	11718	345	919	0.376	1.139	0.999	101.91
p-Coumaric Acid	28140	1667	692	0.344	1.043	0.999	99.89
Vanillin	23701	141	763	0.450	1.365	0.998	101.40
Ferulic Acid	17112	2153	1207	0.988	2.993	0.993	104.54
Hydroxycinnamic acid	36314	-3242	1084	0.452	1.368	0.998	102.16
Rosmarinic Acid	12331	-1814	529	0.601	1.820	0.997	98.45
Quercetin	10757	-2965	493	0.586	1.776	0.998	102.66
Apigenin	14667	-1621	656	0.572	1.733	0.998	99.46
Kampherol	9656	-1047	356	0.471	1.428	0.999	101.87
Hesperitin	15475	-1047	665	0.423	1.112	0.998	99.69
5,7 Dihydroxyflavone (Chrysin)	12818	1916	365	0.399	1.208	0.999	99.21
Rutin	30296	870	751	0.375	1.135	0.999	101.64

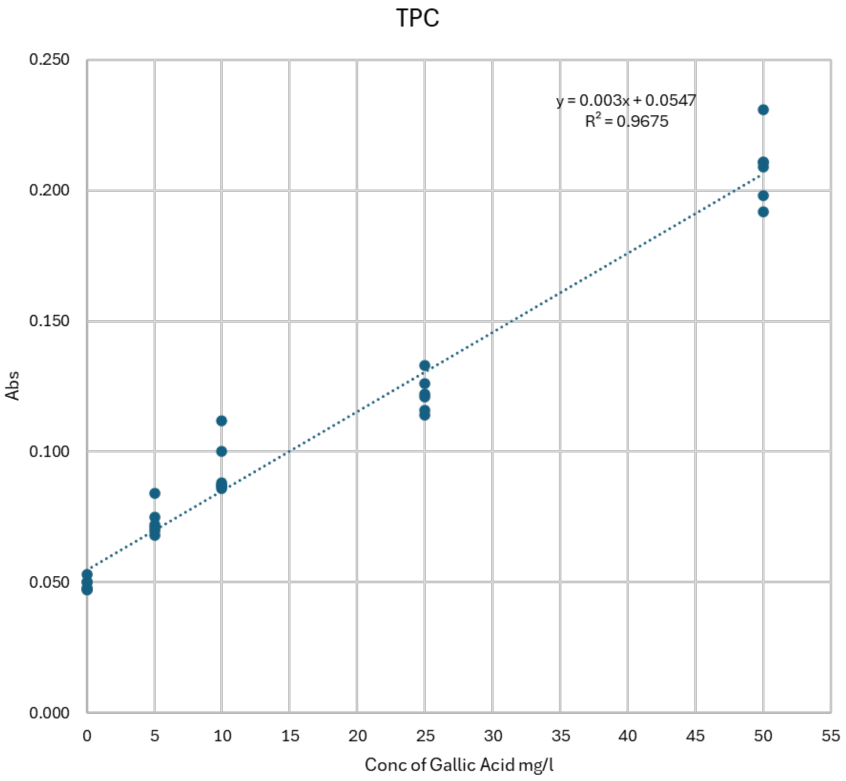

Figure S1: Calibration Curve for the determination of Total Phenolic Content (TPC)

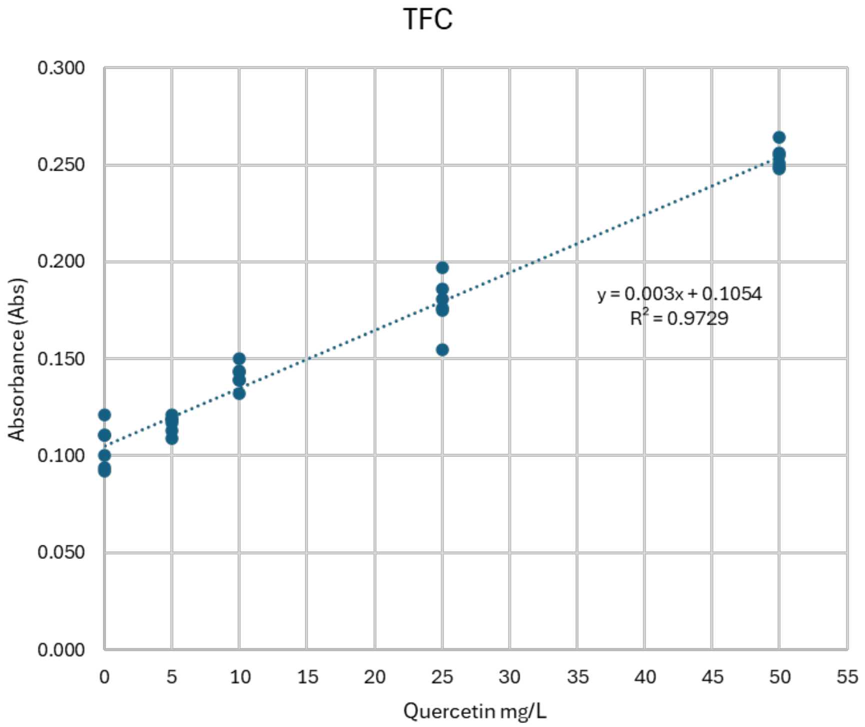

Figure S2: Calibration Curve for the determination of Total Flavonoid Content (TFC)

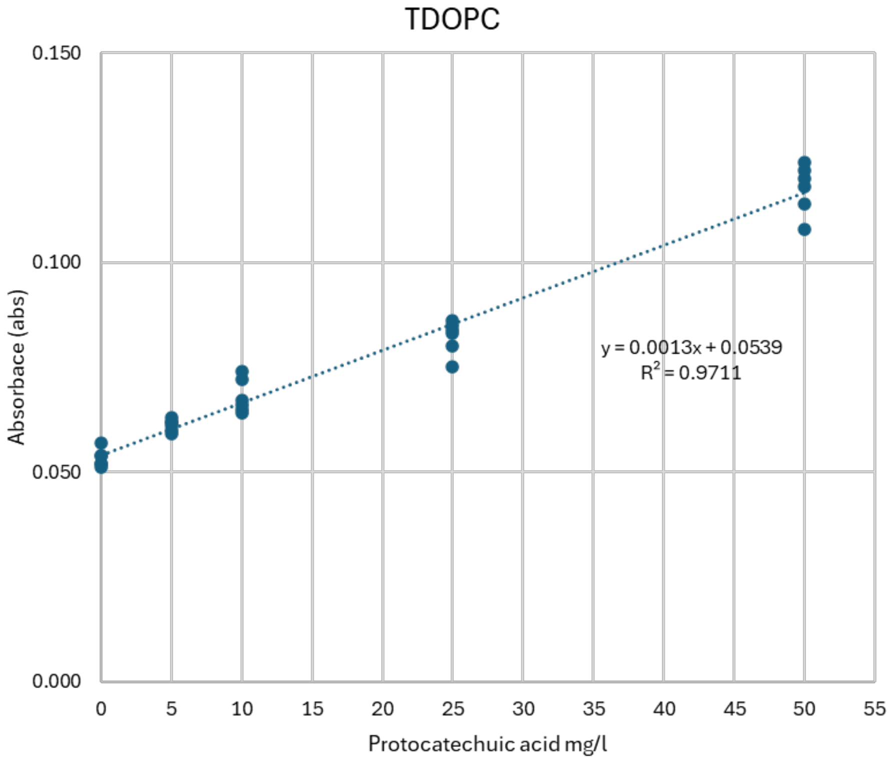

Figure S3: Calibration Curve for the determination of Total Orthodiphenolic Content (TdOPC)

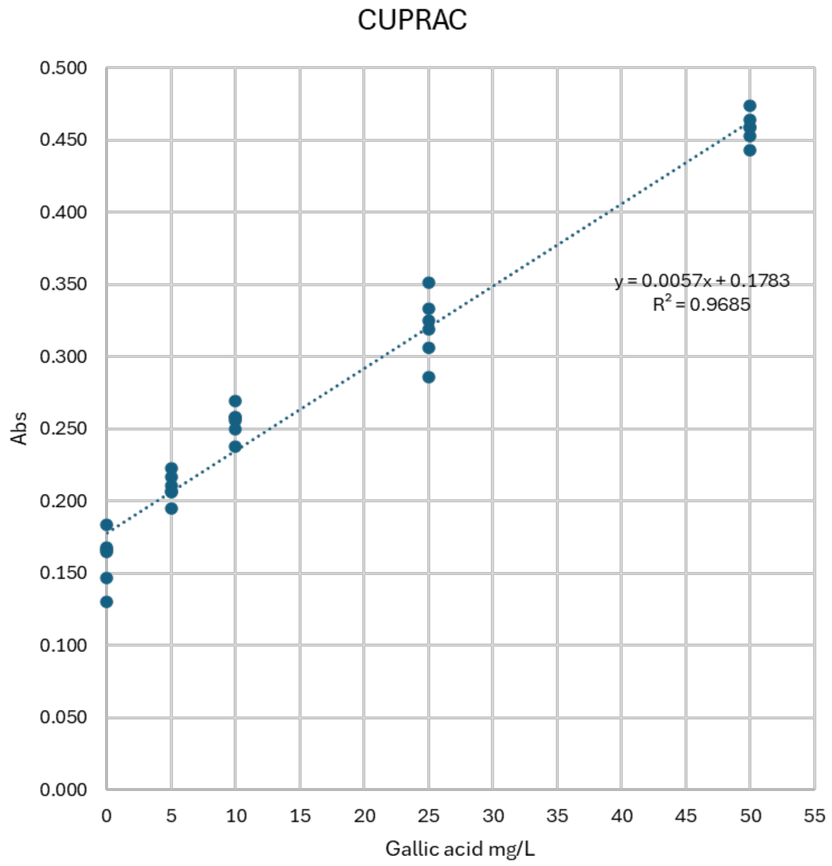

Figure S4: Calibration Curve for the determination of CUPRAC Antioxidant activity

Table S2: LOD and LOQ for the chromometric assays

	**TPC**	**TFC**	**TdOPC**	**CUPRAC**
LOQ (mg/L)	8.827	8.038	8.313	8.694
LOD (mg/L)	2.913	2.653	2.743	2.869
Recovery	0.97	0.956	0.98	0.98

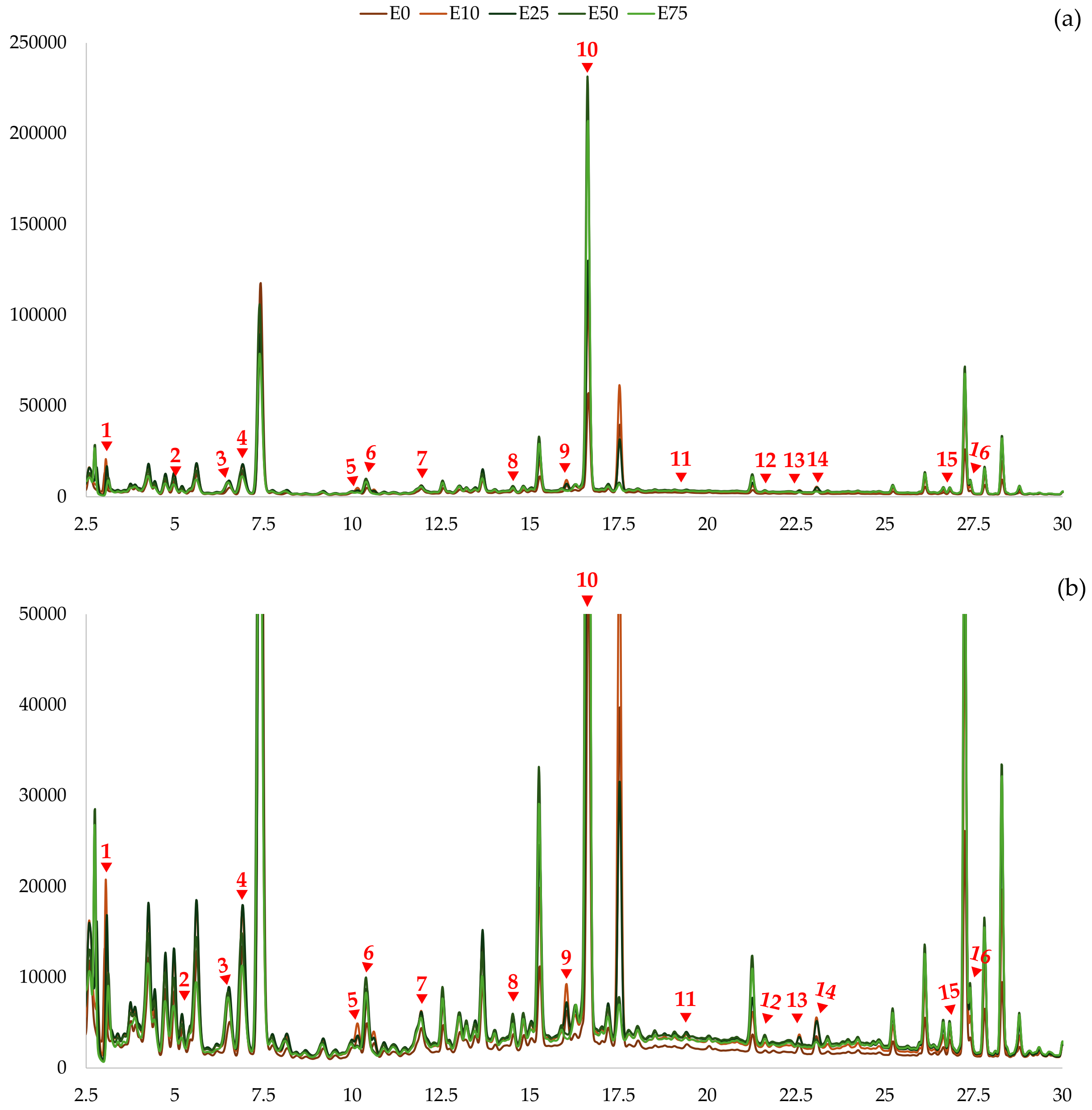

Figure S5: Example of chromatograms with variation in Ethanol concentration: (a) full-scale and (b) zoomed-in HPLC-UV chromatograms of the phenolic compounds in UACPE extracts with various ethanol concentrations. Annotations: (1) Gallic acid, (2) Chlorogenic acid, (3) Caffeic acid (4) Vanillic acid, (5) p-Coumaric acid, (6) Vanillin, (7) Ferulic acid, (8) 2-hydroxyccynnamic acid, (9) Rosmarinic acid, (10) Hesperidin, (11) Quercetin, (12) Apigenin, (13) Kaempferol, (14) Hesperetin, (15) Chrysin, (16) Rutin.

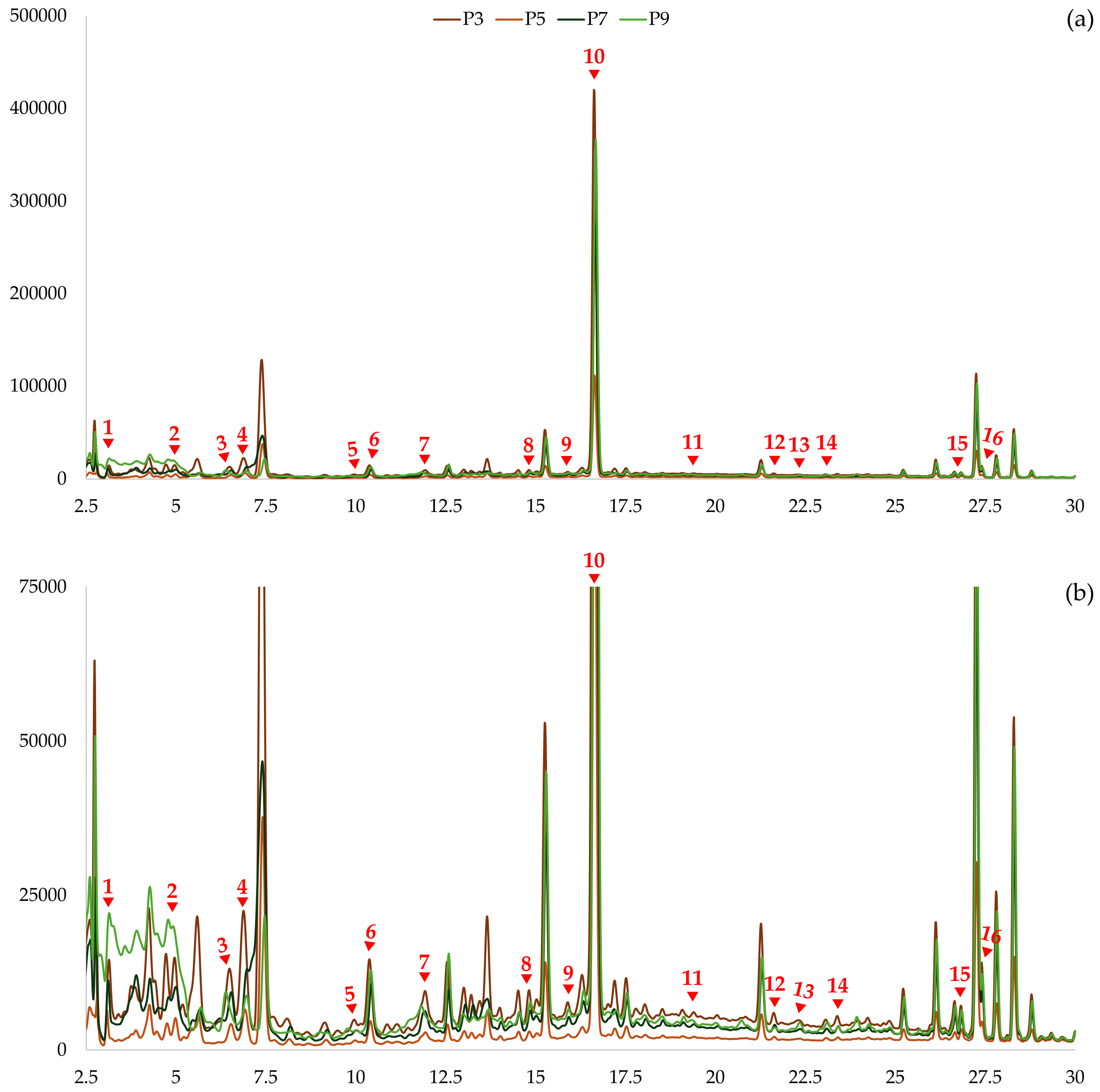

Figure S6: Example of chromatograms with variation in pH of extraction medium: (a) full-scale and (b) zoomed-in HPLC-UV chromatograms of the phenolic compounds in UACPE extracts with various ethanol concentrations. Annotations: (1) Gallic acid, (2) Chlorogenic acid, (3) Caffeic acid (4) Vanillic acid, (5) p-Coumaric acid, (6) Vanillin, (7) Ferulic acid, (8) 2-hydroxyccynnamic acid, (9) Rosmarinic acid, (10) Hesperidin, (11) Quercetin, (12) Apigenin, (13) Kaempferol, (14) Hesperetin, (15) Chrysin, (16) Rutin.

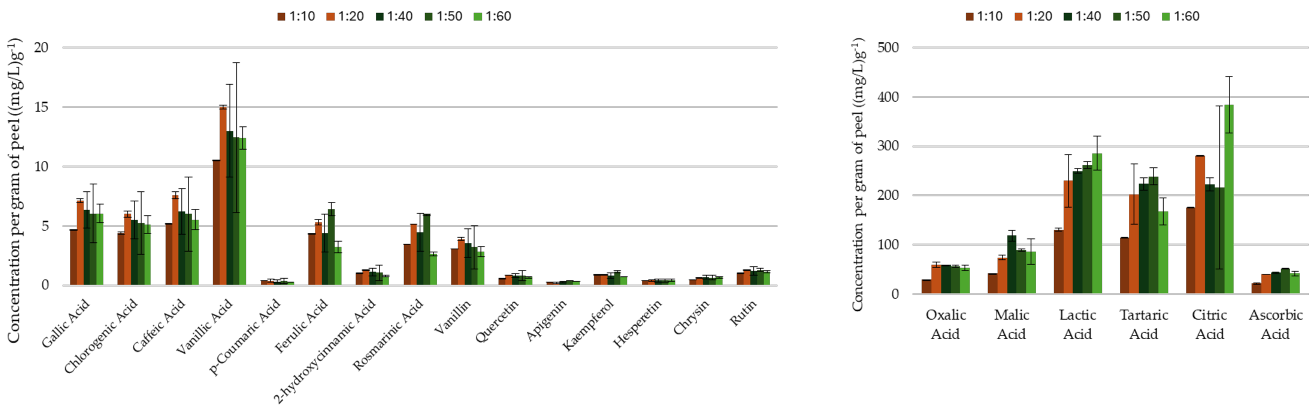

Figure S7: Effect of solid (peel) to liquid (solvent) ratio on the concentrations of phenolic compounds and organic acids (legend units = g/mL)

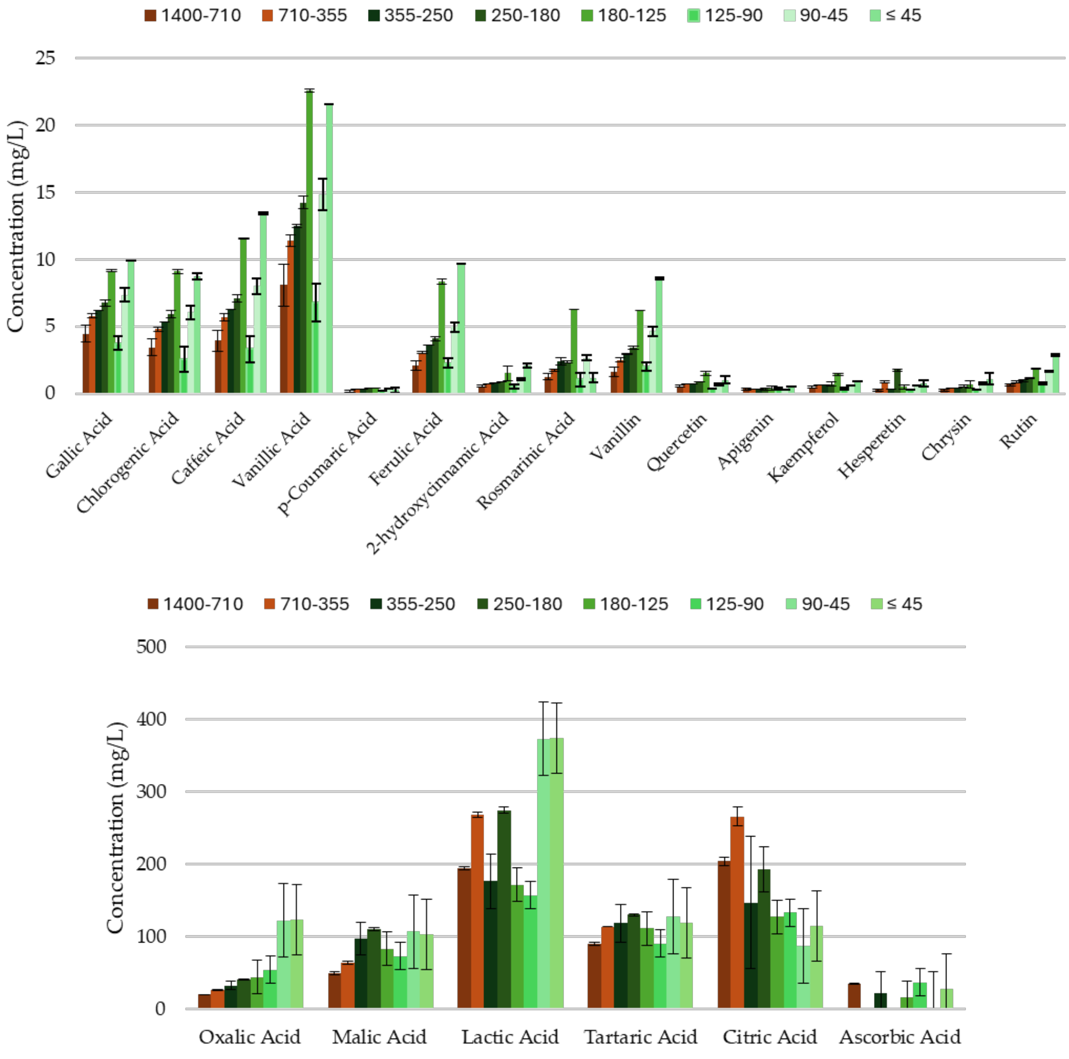

Figure S8: Effect of particle size on the concentrations of phenolic compounds and organic acids (legend units = μm)

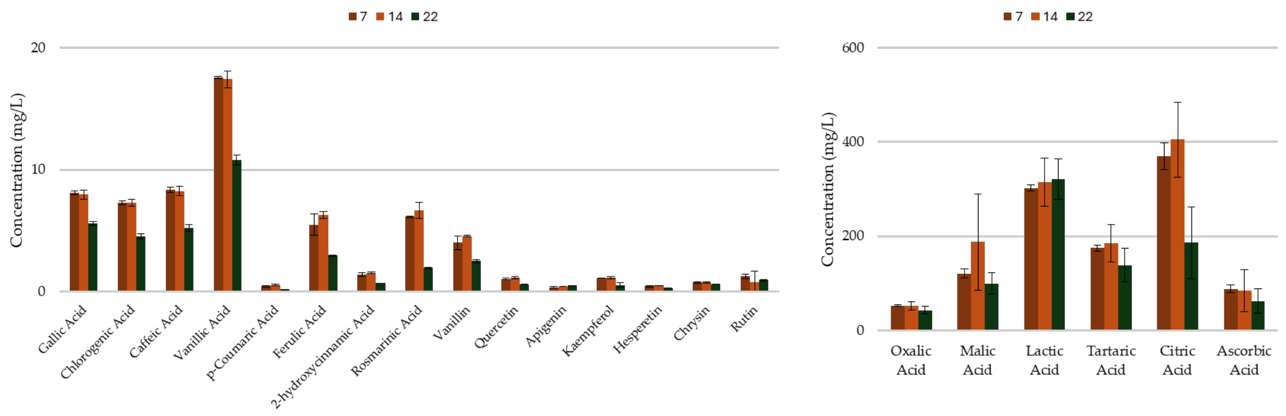

Figure S9: Effect of probe size diameter on the concentrations of phenolic compounds and organic acids (legend units = mm)

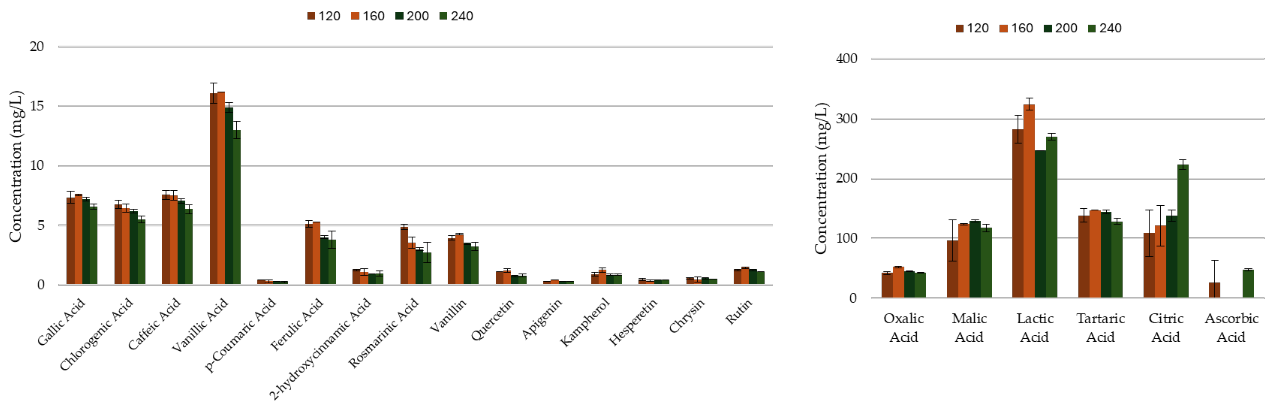

Figure S10: Effect of probe immersion depths on the concentrations of phenolic compounds and organic acids (legend units = mm)
